# Ribosomes binding to *TAS* transcripts buffer ta-siRNA biogenesis in *Arabidopsis thaliana*

**DOI:** 10.3389/fpls.2025.1561041

**Published:** 2025-05-19

**Authors:** Han Wang, Qiming Wen, Minglei Zhao, Hualong He, Jie Cui, Chenjiang You

**Affiliations:** ^1^ State Key Laboratory of Genetic Engineering and Ministry of Education Key Laboratory of Biodiversity Sciences and Ecological Engineering, Institute of Plant Biology, School of Life Sciences, Fudan University, Shanghai, China; ^2^ Guangdong Provincial Key Laboratory for Plant Epigenetics, College of Life Sciences and Oceanography, Shenzhen University, Shenzhen, China; ^3^ College of Horticulture, South China Agricultural University, Guangzhou, China; ^4^ Guangdong Basic Research Center of Excellence for Precise Breeding of Future Crops, Guangdong Laboratory for Lingnan Modern Agriculture, Guangdong Provincial Key Laboratory for the Development Biology and Environmental Adaptation of Agricultural Organisms, South China Institute for Soybean Innovation Research, College of Life Sciences, South China Agricultural University, Guangzhou, China

**Keywords:** ta-siRNA, *Arabidopsis thaliana*, ribosome, degradome, miRNA

## Abstract

Small RNAs, including ta-siRNAs, play crucial roles in various processes in plants. Efforts have been made for decades to elucidate the biogenesis and function of ta-siRNAs. Though the key proteins involved in ta-siRNA biogenesis have been identified, the subcellular localization where ta-siRNAs are processed remains largely unexplored. Remarkably, non-coding *TAS* transcripts were reported to be bound by ribosomes, the machinery responsible for protein translation. Utilizing edited *TAS* genes in *Arabidopsis*, a combination of sRNA-seq, mRNA-seq, RIP-seq, and degradome-seq was employed to investigate the role of ribosomes in ta-siRNA biogenesis in this study. In the two-hit model, deletion of ribosome-binding regions resulted in a decrease in the abundance of intact *TAS3* transcripts but did not significantly affect ta-siRNAs production or the efficiency of miRNA-guided cleavage. Conversely, the deletion of ribosome-binding regions led to a significant reduction in ta-siRNA abundance without affecting mRNA levels in the one-hit model. These findings indicate that in the two-hit model, ribosomes primarily stabilize *TAS* transcripts, while in the one-hit model, they suppress miRNA cleavage but facilitate subsequent processing. Collectively, this study proposes a model that ribosomes play distinct roles in the one-hit and two-hit models of ta-siRNA biogenesis, and provides a new angle to investigate the tangled connection between small RNAs, including miRNA and ta-siRNA, and translation.

## 1 Introduction

Small RNAs (sRNAs) constitute a category of non-coding RNAs with lengths between 18 and 30 nucleotides (Yu et al., 2019). These molecules play essential roles in regulating plant growth and reproduction, as well as in mediating responses to biotic and abiotic stresses (Zhan and Meyers, 2023). Small RNAs are classified into miRNAs and siRNAs based on differences in their precursors and biogenesis (Axtell, 2013a; Fei et al., 2013; Zhan and Meyers, 2023). MiRNAs, the most extensively studied type of sRNA, are encoded by *MIR* genes and typically have a length of 21 nucleotides (Axtell and Meyers, 2018). These miRNAs can be incorporated into ARGONAUTE proteins (AGOs) to form the RNA-induced silencing complex (RISC) (Rogers and Chen, 2013; Yu et al., 2017; Iwakawa and Tomari, 2022; Zhan and Meyers, 2023). Typically in plants, miRNAs regulate target gene expression at the post-transcriptional level by guiding RISC to cleave target mRNAs (Rogers and Chen, 2013; Yu et al., 2017; Zhan and Meyers, 2023). Following cleavage by miRISC, the majority of transcript fragments are degraded by exonucleases (Gy et al., 2007; Rogers and Chen, 2013). However, a small proportion of cleaved transcripts can be processed into dsRNA, which are subsequently cleaved by the endoribonuclease DICER-LIKE proteins (DCLs), resulting in head-to-tail arranged siRNAs, known as phased secondary siRNAs (phasiRNAs) (Vazquez et al., 2004; Allen et al., 2005). Genomic regions capable of producing phasiRNAs are termed *PHAS* loci, which are categorized as either protein-coding loci or non-coding loci, with the latter also referred to as *TAS* loci (Fei et al., 2013).

The biogenesis of ta-siRNAs begins with the transcription of *TAS* genes by RNA polymerase II (Pol II) (Borges and Martienssen, 2015). The resulting full-length *TAS* transcripts, possessing a 5’ cap and a polyA tail (Borges and Martienssen, 2015), are subsequently transported to the cytoplasm. Two pathways exist for further processing: the one-hit model (Allen et al., 2005) and the two-hit model (Axtell et al., 2006; de Felippes et al., 2017). Among the eight known *TAS* genes from four families in *Arabidopsis*, *TAS1a/b/c* and *TAS2* are triggered by miR173 (Allen et al., 2005; Montgomery et al., 2008b), and *TAS4* is triggered by miR828 to produce ta-siRNAs through the one-hit model (Rajagopalan et al., 2006), while *TAS3a/b/c* triggered by miR390 follow the two-hit model (Montgomery et al., 2008a). In the one-hit model, an AGO1 containing a 22-nucleotide length miRNA (Chen et al., 2010; Cuperus et al., 2010; Li et al., 2016) binds to the *TAS* transcript through sequence complementarity and cleaves it into two fragments. The 3’ end fragment of the *TAS* transcript is retained for further processing, while the 5’ fragment undergoes degradation by exoribonuclease (Souret et al., 2004; Liu et al., 2020). In the two-hit model, *TAS* transcripts experience dual binding by AGO7-RISC complexes (loaded with a 21-nucleotide miRNA) at different loci, with cleavage occurring only at the 3’ end binding site (Axtell et al., 2006; de Felippes et al., 2017). The 5’ end fragment undergoes subsequent processing steps (Axtell et al., 2006). In both models, the retained fragments are processed into double-stranded RNAs (dsRNAs) by RNA-dependent RNA polymerase6 (RDR6), with SUPPRESSOR OF GENE SILENCING 3 (SGS3) protecting fragments from degradation (Peragine et al., 2004; Vazquez et al., 2004; Yoshikawa et al., 2005; Yoshikawa et al., 2013), starting from the 3’ end (Fei et al., 2013). DCL4 then utilizes the dsRNA as a substrate to generate 21-nucleotide ta-siRNAs (Allen et al., 2005; Yoshikawa et al., 2005). These ta-siRNAs are subsequently loaded into AGO1 to silence the target genes.

In addition to DCL4, *Arabidopsis* DCL2, which is primarily associated with the production of natural antisense cis-acting siRNAs (natsiRNAs) (Liu et al., 2009), can also compete with DCL4 to cleave *TAS*-derived dsRNAs, resulting in a small quantity of 22-nt ta-siRNAs (Lu et al., 2022). Similar to 22-nt miRNA triggers, 22-nt ta-siRNAs can target their homologous transcripts as precursors to generate ta-siRNAs (Tamim et al., 2018) subsequently. This process occurs according to the one-hit model, leading to the production of ta-siRNAs with a distinct phase character (Tamim et al., 2018). These ta-siRNAs produced by 22-nt ta-siRNA are termed tertiary sRNAs, which also form functional RISC to cleave transcripts. Given that cleavage consistently occurs between the 10th and 11th nucleotide, counting from the 5’ end of the miRNA or ta-siRNA (Yu et al., 2019; Liu et al., 2020), the phase of ta-siRNA and tertiary sRNA differs by 9 nucleotides (Tamim et al., 2018; Liu et al., 2020).

Ta-siRNAs are involved in various developmental and metabolic pathways in *Arabidopsis*. The *TAS3*-*ARF* module is particularly well-studied within the plant *TAS* network. *AUXIN RESPONSE FACTORS* (*ARFs*) encode a crucial class of plant transcription factors that govern the expression of auxin response genes (Luo et al., 2018). *TAS3*-derived ta-siRNAs suppress the expression of *ARF2*, *ARF3*, and *ARF4*, thereby regulating the plant’s response to auxin (Xia et al., 2017). This regulation influences several developmental processes, including leaf polarity formation (Xu et al., 2007), vegetative phase transition (Fahlgren et al., 2006), lateral root growth (Marin et al., 2010), and female germline specification (Su et al., 2017). Furthermore, *TAS1*-derived ta-siRNAs regulate thermotolerance by targeting the *HEAT-INDUCED TAS1 TARGET* (*HTT*) family (Kume et al., 2010; Li et al., 2014; Zhao et al., 2016). Overexpression of *HTT* upregulates several heat shock factor genes (*HSFs*), enhancing thermotolerance (Li et al., 2014). Additionally, *TAS1* and *TAS2*-derived ta-siRNAs contribute to bolstering plant immune responses during encounters with fungal pathogens (Cai et al., 2018; Padilla-Padilla et al., 2024). Research indicates that ta-siRNAs derived from *TAS1a*, *TAS1b*, *TAS1c*, and *TAS2* are elevated during *Botrytis cinerea* infections (Padilla-Padilla et al., 2024). These ta-siRNAs are transmitted to fungal cells through extracellular vesicles, suppressing the expression of fungal genes and thereby reducing pathogenicity (Cai et al., 2018). Moreover, *TAS4*-derived ta-siRNAs respond to sugars and target the *R2R3*-*MYB* transcription factors *PAP1*, *PAP2*, and *MYB113* at various leaf developmental stages to regulate anthocyanin production (Luo et al., 2012).

The subcellular localization patterns of miRNA synthesis, transport, and function have been gradually elucidated (Yu et al., 2017). However, the subcellular localization processes of ta-siRNAs remain largely unexplored. One hypothesis suggests that ta-siRNA biogenesis occurs on the rough endoplasmic reticulum (rER), where ribosomes are bound (Li et al., 2016; Liu et al., 2020). Both 22-nt miRNAs and AGO1, crucial triggers of ta-siRNA biogenesis, are enriched and co-fractionated with polysome fractions (Lanet et al., 2009; Brodersen et al., 2012; Li et al., 2013). The relatively weak association of AGO1 with membrane components indicates that AGO1 is a peripheral membrane protein (Brodersen et al., 2012). Similarly, AGO7, the ta-siRNA trigger factor in the two-hit model, is reported to associate with the rER, as evidenced by the presence of the resident ER marker calnexin in the fraction with the highest HA-AGO7 signal (Jouannet et al., 2012). 5’ RACE RT-PCR analysis of 3’ cleavage fragments from miRNA target transcripts reveals that miRNA cleavage of target mRNAs occurs in both membrane-associated polysomes (MBPs) and free polysomes (FPs) (Li et al., 2016). Notably, Ribo-seq evidence demonstrates that non-coding genes *TAS1a/b/c*, *TAS2*, and *TAS3a* all contain small ORFs with ribosome binding in non-ta-siRNA generation regions (Hou et al., 2016; Li et al., 2016). In *Nicotiana benthamiana*, modifying the length and position of the *TAS3a* sORF of *Arabidopsis* results in reduced *TAS3a* transcript levels. Additionally, cordycepin treatment and subsequent decay rate monitoring in mutants and wild-type plants reveal lower stability of *TAS3a* transcripts in mutants compared to wild-type plants (Bazin et al., 2017). In maize, deletion of the ribosome binding site of *24PHAS_NO296* leads to decreased phasiRNA production, while transcript abundance remains unchanged between mutants and wild-type plants (Han et al., 2024). However, direct *in vivo* evidence is still lacking regarding whether ta-siRNA biogenesis in *Arabidopsis* depends on ribosomes, what specific role ribosomes play in this process, and whether there are functional differences in ribosome involvement between the one-hit and two-hit models.

This study investigates the role of ribosomes in the two-hit and one-hit models of ta-siRNA biogenesis in *Arabidopsis*. We generated CRISPR mutants *tas3a/b/c*, which eliminated most ribosome binding regions on *TAS3a*, *TAS3b*, and *TAS3c*, as well as mutants *tas2-1*, *tas2-2*, and *tas2-3*, which deleted varying lengths of ribosome binding regions on *TAS2*. Utilizing high-throughput sequencing methods including sRNA-seq, mRNA-seq, RNA Immunoprecipitation sequencing (RIP-seq), and degradome-seq, we uncovered distinct functions of ribosomes in the one-hit and two-hit models. In the *tas3a/b/c* mutant, the absence of ribosome binding regions resulted in decreased sRNA and mRNA abundance. Analysis of miRNA-guided cleavage efficiency relative to degradome-seq reads at the miRISC cleavage site and total transcript abundance revealed that miR390-guided cleavage efficiency of *TAS3a* transcripts remained unaffected by the loss of these regions. In *tas2* mutants, both ta-siRNA and mRNA abundances decreased proportionally to the extent of *TAS2* ribosome binding region deletion. Notably, the *tas2–3* mutant, with the most extensive deletion of the *TAS2* ribosome binding region, exhibited significantly higher miR173-RISC cleavage efficiency of *TAS2* transcripts compared to the WT. Based on these findings, we propose a model for ribosome function in ta-siRNA biogenesis. In both models, ribosomes protect intact *TAS* transcripts from degradation. In the one-hit model, ribosomes suppress miRNA-mediated cleavage of *TAS* transcripts, while subsequent processes such as dsRNA biogenesis and DCL4-mediated cleavage require a ribosome-rich environment. In contrast, the two-hit model suggests that ribosomes may primarily function to stabilize *TAS* transcripts without additional roles.

## 2 Results

### 2.1 The deletion of ribosome binding regions of *tas3a/b/c* leads to diminished fertility

**Figure 1:**
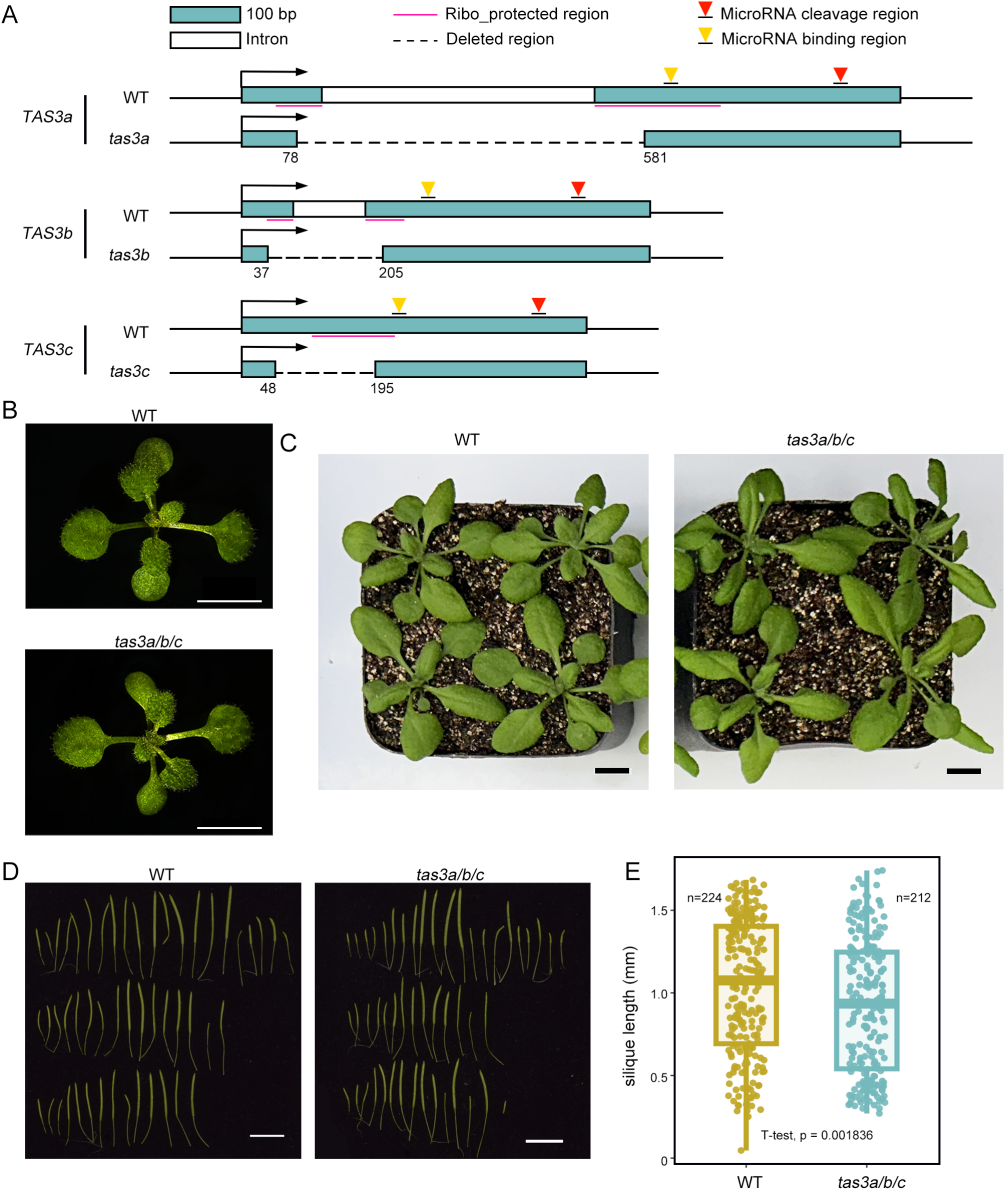
*tas3a/b/c* plants show reduced silique length compared to wild type. **(A)** Diagrams showing CRISPR-mediated deletion of the ribosome binding regions in *TAS3a*, *TAS3b*, and *TAS3c*. **(B)** 12-days-old plants exhibit no significant phenotypical differences between WT and *tas3a/b/c*. Scale bar = 5 mm. **(C)** The 26-days old *tas3a/b/c* plants displayed a slightly downward-curling of leaf margins, compared to WT. Scale bar = 1 cm. **(D)** *tas3a/b/c* showed more fully or partially sterile siliques than WT plants. Scale bar = 1 cm. **(E)** Box plot of siliques length statistics of WT and *tas3a/b/c*.(p<0.05, T-test).

To examine the dependency of the two-hit model of ta-siRNA biogenesis on ribosomes, we engineered truncated mutants of *Arabidopsis TAS3* using CRISPR/Cas9 in *Arabidopsis thaliana*. The targeted deletions were implemented in nucleotides 78–581 of *TAS3a*, 37–205 of *TAS3b*, and 48–195 of *TAS3c*, encompassing the majority of the ribosome binding regions of the *TAS3* gene family while preserving the miR390 binding sites (Axtell et al., 2006) (**Figure 1A**). Subsequently, we analyzed the *TAS3* transcripts in the transgenic plants using mRNA-seq. As anticipated, *TAS3a*, *TAS3b*, and *TAS3c* displayed the designed nucleotide deletions (**Supplementary Figure S1A-C**).

Previous studies have demonstrated that defects in ta-siRNA biogenesis can lead to narrow pointed leaves (referred to as the ‘*zip*’ phenotype) during early vegetative development in the *Arabidopsis* Col-0 ecotype (hereafter referred to as WT) (Gasciolli et al., 2005). However, seedlings of the CRISPR mutant, designated as *tas3a/b/c*, cultivated on 1/2 MS medium for 12 days exhibited no discernible phenotypic differences from the WT (**Figure 1B**). In plants grown in soil for 26 days, a proportion of leaves in *tas3a/b/c* mutants displayed slightly downward-curling leaf margins (**Figure 1C**), a characteristic phenotype consistent with previously characterized ta-siRNA biogenesis defective mutants *ago7* and *dcl4*, which may be due to the ectopic and increased expression of *ARFs* (Adenot et al., 2006; Jouannet et al., 2012) (**Supplementary Figure S1D**). *TAS3*-derived ta-siRNAs also play a crucial role in *Arabidopsis* reproductive development (Su et al., 2017). After 40 days of growth, a statistical analysis was conducted on the lengths of all siliques from *tas3a/b/c* and WT plants, with five plants each. The average length of the mutants’ siliques was 0.89 cm, significantly shorter than that of the WT, which measured 1.02 cm (P value = 0.001836, **Figure 1D, E**).

Thus, the phenotype of *tas3a/b/c* bears resemblance to, yet differs from, those of reported ta-siRNA biogenesis defective mutants. This suggests that while the shortened ribosome-binding regions impact ta-siRNA biogenesis, *TAS3*-derived ta-siRNAs may still persist in the mutants.

### 2.2 Tertiary ta-siRNAs are absent in *tas3a/b/c*

**Figure 2:**
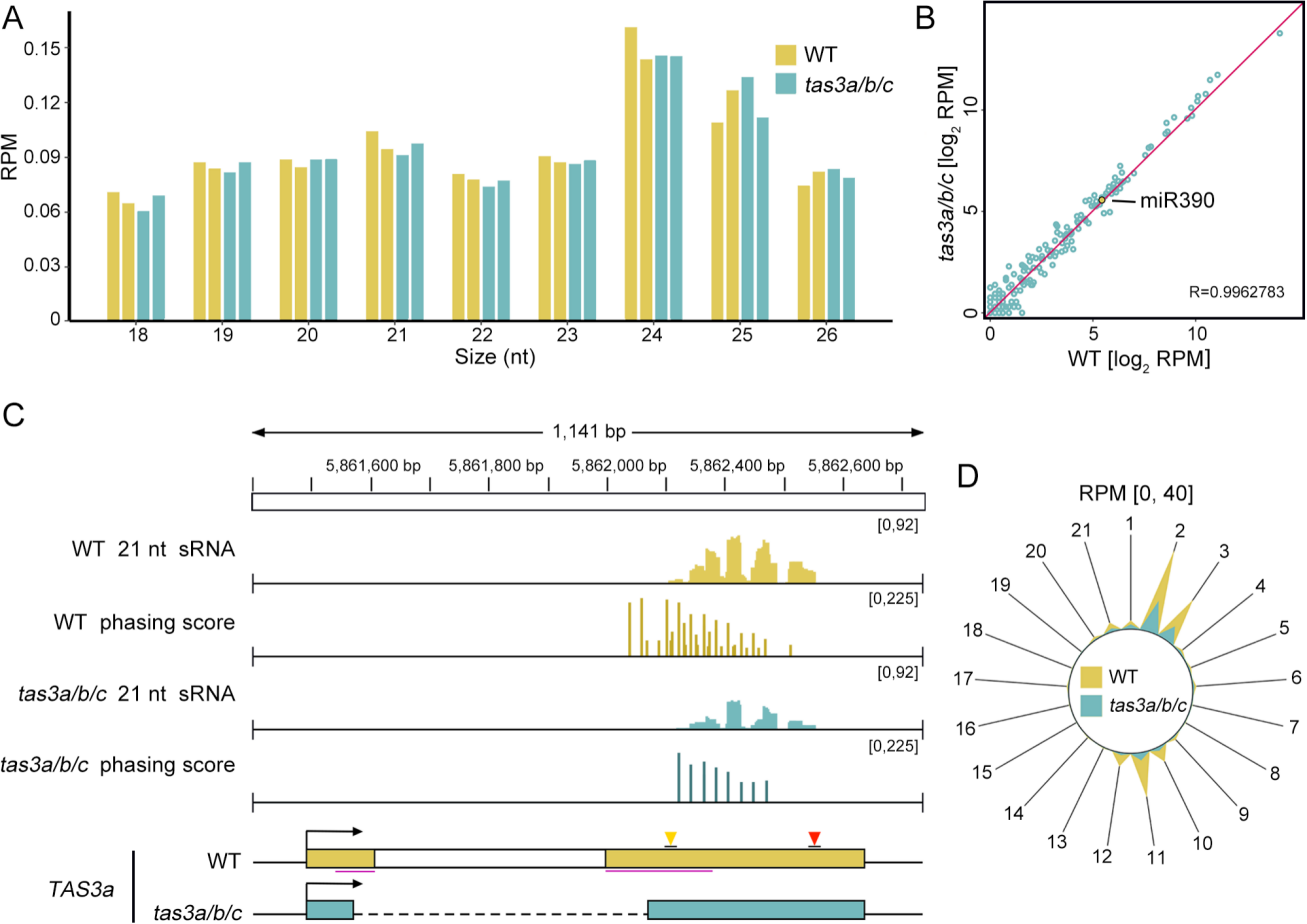
The levels of tas3a/b/c sRNAs are decreased, while the tertiary sRNAs are nearly disappeared. **(A)** Length distribution of sRNAs in WT and *tas3a/b/c* mutants. The y-axis represents the abundance (reads per million, RPM) of sRNAs of varying lengths. **(B)** A scatter plot showing comparable miRNA abundance in *tas3a/b/c* and WT. **(C)** IGV display illustrates the abundance and phasing score of 21-nt sRNAs at the *TAS3a* locus in WT and *tas3a/b/c* mutant plants. The y-axis range for each track is indicated in the upper right corner. In the phasing score tracks, the high phasing score loci represent ta-siRNAs generated by the miR390 trigger, while the short bars between these high bars represent tertiary sRNAs produced by the 22-nt ta-siRNAs triggering *TAS3* transcripts. Notably, compared to WT, the phasing score of tertiary sRNAs in *tas3a/b/c* mutants is significantly reduced. **(D)** The radial graph illustrates the abundance of sRNAs at various phases. The polar coordinate axis represents the reads abundance (RPM) of the 5’ end falling into this phasing register, with values ranging from 0 to 40. The peak at spoke 2 indicates the abundance of ta-siRNAs generated by the miR390 trigger, while the peak at spoke 11 signifies tertiary sRNAs. All radial graphs presented in this article are derived from the average of two replicates.

To assess the effect of ribosome-binding-region deletion on ta-siRNA biogenesis from *TAS3*, we performed sRNA sequencing in both *tas3a/b/c* and WT seedlings. Initially, we analyzed the length distribution and landscape of reads aligned to the genome as a quality control measure. Consistent with previous studies (Borges and Martienssen, 2015), 24-nt sRNA was significantly more abundant than other lengths, with 21-nt sRNA being the second most prevalent in all WT and *tas3a/b/c* samples (**Figure 2A**). To rule out the possibility that changes in ta-siRNA biogenesis were due to altered miRNA expression, we examined the abundance (Reads per million mapped reads, RPM) of all miRNAs, particularly miR390, in *tas3a/b/c*. The results indicated that miRNA expression remained unaffected in the mutant (**Figure 2B**).

Subsequently, the abundance of small RNAs (sRNAs) from all three members of *TAS3*, namely *TAS3a*, *TAS3b*, and *TAS3c*, was evaluated using ShortStack, a tool that allocates sRNAs mapped to multiple genomic loci based on neighboring sRNA coverage (Shahid and Axtell, 2014). The analysis revealed a drastic reduction in sRNA abundance derived from all three genes in the *tas3a/b/c* mutant (**Supplementary Figure S1E**). Notably, sRNAs originating from *TAS3b* and *TAS3c* were detected in minimal quantities in the WT plants (**Supplementary Figure S1B, C**), suggesting that the majority of *ArabidopsisTAS3*-derived trans-acting siRNAs (ta-siRNAs) originated from *TAS3a*. Consequently, subsequent analyses focused exclusively on *TAS3a*.

Ta-siRNAs constitute a distinct class of siRNAs characterized by a head-to-tail pattern during their biogenesis. To quantify this pattern and abundance of ta-siRNAs at specific loci, we employed the phasing score as a metric (Yang et al., 2018). The calculation of the phasing score at any 21-nt ta-siRNA generating locus, specifically the DCL4-processing site, utilized a sliding window approach. This method progressed along the genome in 1-nt increments from the 5’ to the 3’ end. Within an 189bp window meeting the coverage threshold, each nucleotide was assigned to a register, numbered 1 to 21 (resetting to 1 after reaching 21). Nucleotides sharing the same register number indicate the same phase of DCL4-mediated cleavage. The phasing score for each register was calculated as described in (Yang et al., 2018), with an elevated phasing score signifying that a particular coordinate generates a high abundance of orderly arranged ta-siRNAs.

At the *TAS3a* locus, the sRNA abundance decreases in the *tas3a/b/c* mutant compared to the WT (**Figure 2C**). Upon analysis of phasing scores on this transcript, we identified only 13 coordinates within *TAS3a* exhibiting phasing scores above threshold in *tas3a/b/c*, while 38 coordinates in WT were notable (**Figure 2C**). A Wilcoxon test comparing the 13 phasing scores of the *tas3a/b/c* to those at corresponding positions in the WT revealed no significant difference (P value = 0.5114) between the two samples. This suggests that the decrease in sRNA abundance in *tas3a/b/c* may be attributable to registers losing phasing scores. Considering the potential cascade biogenesis of *TAS3a* ta-siRNAs, we attempted to separate different phases by consolidating all siRNAs from each phase using registers. Consequently, siRNAs aggregated from each register were depicted in a radial graph (**Figure 2D**). By determining the distance between the miR390-guided cleavage site and the nearest DCL4 cleavage site, we identified the primary phase at register 2 and the secondary phase triggered by 22-nt secondary ta-siRNAs at register 11. Intriguingly, tertiary ta-siRNAs from register 11 exhibited a greater decrease compared to secondary ones from register 2.

This alternation is also evident in the IGV representation of the *TAS3a* phasing scores (**Figure 2C**). In WT, the distribution of phasing scores exhibited a short bar flanked by two taller bars, with the short bars indicating register 11 and the taller bars representing registers 2 and 3. Notably, the short bars are absent in *tas3a/b/c*. Collectively, our findings suggest that while the biogenesis of secondary ta-siRNAs remains unaffected by the deletion of the ribosome binding regions, the mutation significantly impacts the biogenesis of tertiary ta-siRNAs.

### 2.3 22nt-ta-siRNA could be properly loaded into AGO1 in *tas3a/b/c*

The biogenesis of tertiary siRNA at the *TAS3a* locus initiates with the incorporation of 22-nt *TAS3*-derived ta-siRNAs into AGO1, followed by the subsequent cleavage of *TAS3a* transcripts. Despite the deletion of the majority of ribosome binding regions in *TAS3a*, *TAS3b*, and *TAS3c*, the *tas3a/b/c* mutant can still produce secondary *TAS3*-derived ta-siRNAs, suggesting that the biogenesis of 22-nt *TAS3*-derived ta-siRNA in this mutant might be ribosome-independent. Given that AGO1 co-localizes with ribosomes (Lanet et al., 2009; Brodersen et al., 2012; Li et al., 2013), the subcellular localization discrepancy between ta-siRNA and AGO1 could potentially impede the formation of 22-nt ta-siRNAs RISC. To examine this hypothesis, we conducted RIP-seq to sequence AGO1-bound small RNAs from both the *tas3a/b/c* mutant and WT plants, thereby assessing their loading efficiency.

**Figure 3:**
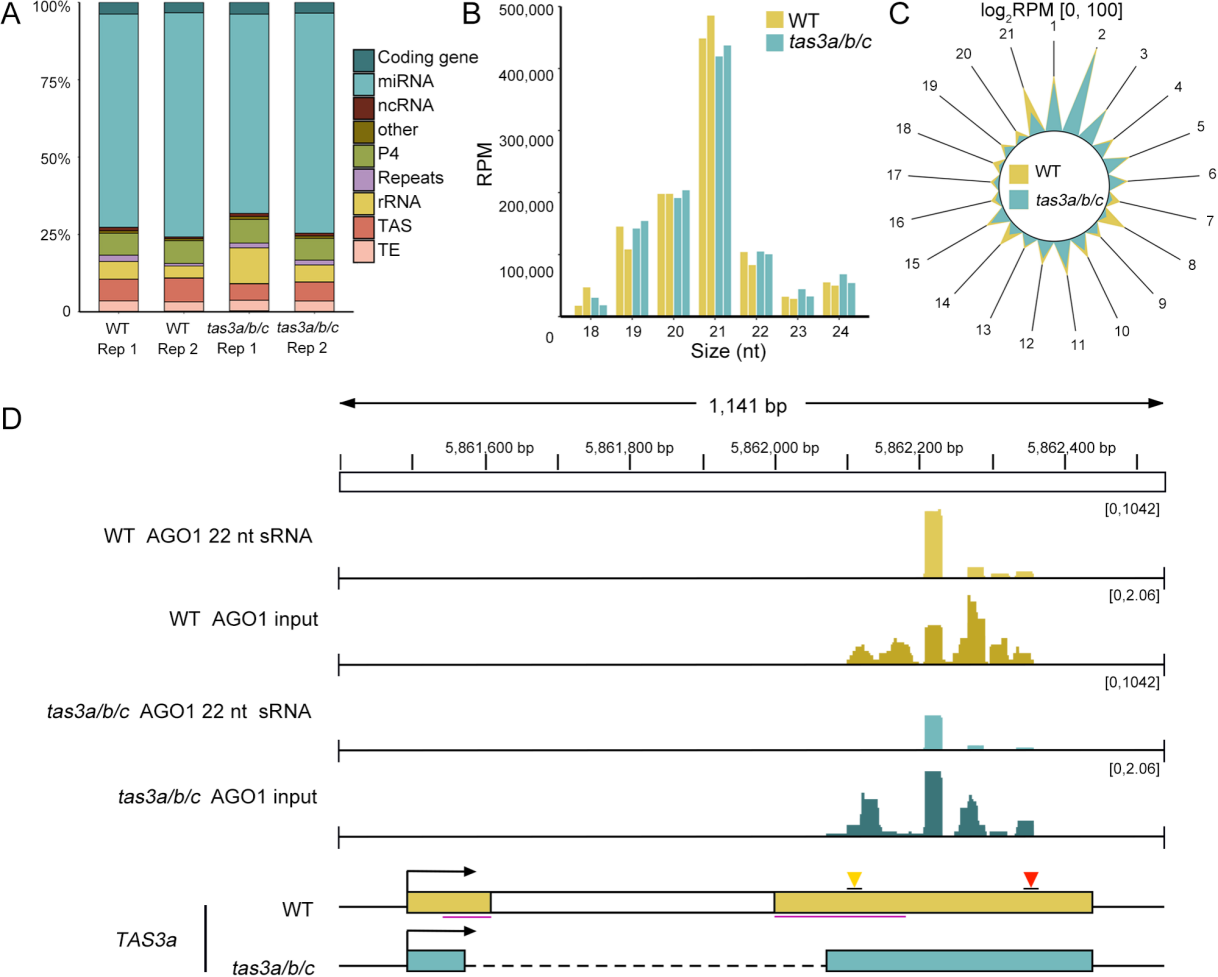
The loading of 22-nt ta-siRNAs into AGO1 remains unchanged. **(A)** Classification of genomic features of sRNAs loading into AGO1 in WT and *tas3a/b/c* mutants. **(B)** Length distribution of sRNAs in WT and *tas3a/b/c* mutants loading into AGO1. Consistent with previous studies, the length of sRNA associated with AGO1 is predominantly 21 nucleotides. **(C)** The radial graph illustrating the abundance (RPM) of sRNAs at different phases loading into AGO1. **(D)** IGV display of the abundance of 22-nt ta-siRNAs associated with AGO1, with total sRNAs as input. Despite a slight reduction in quantity, 22-nt ta-siRNAs maintain effectively loading into AGO1.

An analysis of sRNA species and length distribution from the AGO1 RIP-seq data was initially performed. In alignment with previous research (Liang et al., 2023), our dataset demonstrated that miRNAs constitute the majority of sRNA species associated with AGO1 (**Figure 3A**). Furthermore, the analysis revealed that the predominant size of sRNAs in AGO1 is 21 nt (**Figure 3B**).

The radial graph of *TAS3a*-derived AGO1-bound siRNAs demonstrated that register 2 was the predominant phase (**Figure 3C**), which aligns with the findings from the total sRNA-seq (**Figure 2D**). Importantly, the register-2 peak in *tas3a/b/c* remained comparable to that in WT (**Figure 3C**), and the abundance of 22-nt ta-siRNA precipitated with AGO1 exhibited only a modest reduction while remaining highly abundant in *tas3a/b/c* (**Figure 3D**). Thus, the absence of ribosome binding regions may not significantly impair the biogenesis and loading of 22-nt ta-siRNA into AGO1.

### 2.4 *TAS3a* transcripts undergo normal miRISC-mediated cleavage in *tas3a/b/c*

**Figure 4:**
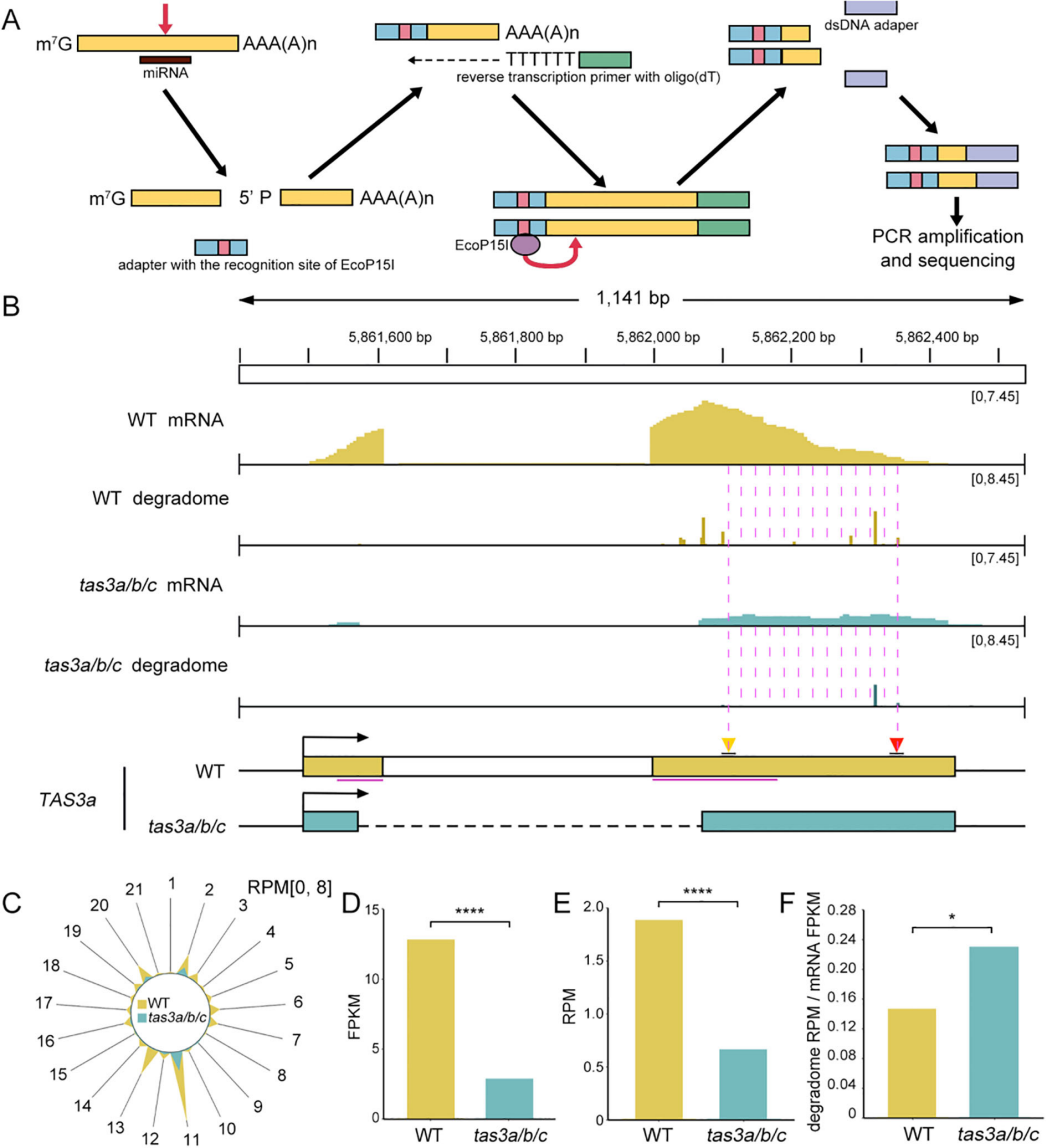
The cleavage efficiency of *TAS3a* transcripts in the *tas3a/b/c* mutant is minimally affected. **(A)** Schematic representation of degradome library construction. Following miRNA-mediated cleavage, the mRNA is separated into 5’ and 3’ fragments, with the latter possessing a 5’ monophosphate. The 3’ fragment is ligated to a 5’ adapter containing an EcoP15I restriction site. Reverse transcription is conducted using an oligo(dT) primer to isolate the adapter-ligated poly(A) RNA. Subsequently, EcoP15I digestion is employed to extract the 5’ cleavage site, followed by 3’ adapter ligation to the resulting 5’ fragments for PCR amplification. Sequencing of the amplified products reveals the precise locations of miRNA-induced cleavage sites. **(B)** The IGV display illustrates the abundance of full-length and cleaved transcripts at the *TAS3a* locus in WT and *tas3a/b/c* mutants. The quantity of cleaved transcripts at a particular site is determined by counting the number of reads whose 5’ end aligns with the site, expressed in RPM. The distance between the backgrounds dashed lines represents 21 nucleotides. **(C)** The radial graph illustrates sRNA-mediated cleavage events on the *TAS3a* locus, depicting the frequency of these events across various phases. The RPM is computed based on the alignment of the 5’ end with each register. Spoke 2 specifically represents cleavage mediated by miR390. **(D)** Full-length transcript abundance (FPKM) of *TAS3a* in WT and *tas3a/b/c*, with FPKM values representing the average of those in two replicates. *P* < 0.0001. * represents *P* < 0.05, **** represents *P* ≤ 0.0001. **(E)** Abundance of transcripts cleaved by miR390-AGO7 in WT and *tas3a/b/c* mutants. The RPM values are calculated based on the alignment of the 5’ end mapping to the position chr3:5862354, with the RPM values representing the average of two biological replicates. Fisher’s exact test *P* < 0.0001. **(F)** The cleavage efficiency of miR390 on *TAS3a* transcripts in WT and *tas3a/b/c* mutants (ratio of cleaved transcript abundance **(E)** to full-length transcript abundance **(D)**). One-sided t-test *P* = 0.0383.

To investigate whether the subsequent stage in tertiary siRNA biogenesis was affected by the manipulation of ribosome binding regions, we conducted degradome sequencing in both *tas3a/b/c* and WT. Degradome sequencing, also known as parallel analysis of RNA ends (PARE) (Addo-Quaye et al., 2008; German et al., 2008), is a technique that enables genome-wide validation of miRNA targets. This method capitalizes on the fact that miRNA-guided cleavage produces an uncapped 5’ end on the 3’ fragment, allowing the ligation of a 5’ adapter containing an EcoP15I endonuclease recognition site. Subsequently, oligo(dT) primers are used for reverse transcription to synthesize double-stranded cDNA. The EcoP15I endonuclease then cleaves 25 nucleotides downstream of the recognition site. These products are ligated to a 3’ adapter, PCR amplified, and sequenced (**Figure 4A**). By aligning degradome reads to the *Arabidopsis* genome and focusing on the 5’ end counts, we compared targeted cleavage on *TAS3* transcripts between WT and *tas3a/b/c* mutant.

The miR390-guided cleavage (indicated by the red triangle) at the *TAS3a* locus was observed to be reduced in the *tas3a/b/c* mutants (**Figure 4B**). Analysis of the radial plot revealed that register 2, representing the miR390-triggered cleavage that generates ta-siRNAs (**Figure 4C**), was depleted in the mutant. This suggests that the deletion of the ribosomal binding region impacted the miRNA-guided cleavage. Consequently, secondary siRNAs, which triggered cis-cleavage of *TAS* transcripts and generated tertiary siRNAs, also showed reduced abundance in the corresponding register 20 (**Figure 4C**).

This observation led to a subsequent inquiry: whether the reduction of miRISC cleavage is attributable to the absence of ribosomes bound to edited *TAS* transcripts or the decrease of *TAS3* transcripts in *tas3a/b/c*. Although *TAS* genes are non-coding, they possess typical transcript features such as a 5’ m7G cap and 3’ polyA tail. Accordingly, we conducted mRNA-seq to examine the abundance of *TAS3a* primary transcripts. The results revealed that the abundance of *TAS3* primary transcripts in *tas3a/b/c* was significantly reduced compared to the WT (**Figure 4D**). However, next-generation mRNA-seq primarily detects intact transcripts, disregarding cleaved fragments lacking polyA tails. Thus, the reduction in transcript abundance could be attributed to either the instability of intact transcripts or accelerated processing. To address this, we utilized degradome reads abundance(RPM) at the miR390 cleavage site(**Figure 4E**) in comparison with the abundance(FPKM) of *TAS3a* full length transcripts to calculate the impact of deleting the ribosome binding region on miR390 cleavage efficiency. Although the abundance of both intact transcripts and miRISC-cleaved transcripts of *TAS3a* decreased in *tas3a/b/c* (**Figure 4D, E**), the processing efficiency of miR390-guided cleavage (degradome RPM/mRNA FPKM) was actually elevated (**Figure 4F**).

In conclusion, these results demonstrate that ribosome binding to transcripts suppresses miRNA-guided cleavage in the two-hit model. However, the impact of ribosomes on tertiary siRNA biogenesis remains unclear, primarily due to the impaired production of secondary siRNAs.

### 2.5 21-nt ta-siRNAs derived from *TAS2* exhibit reduced accumulation in *tas2* mutants

**Figure 5:**
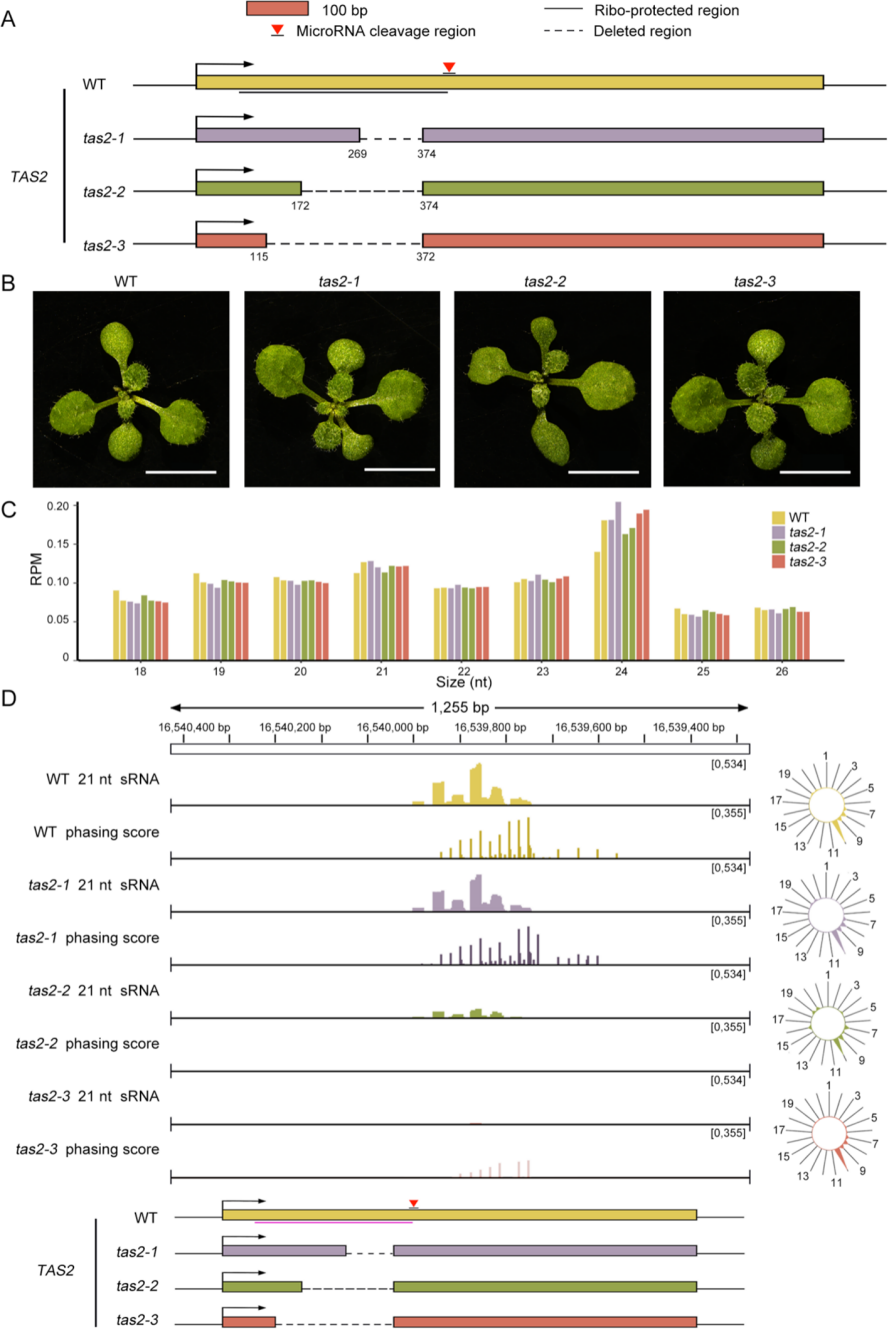
21-nt sRNAs abundance decreases as the ribosome binding regions shorten. **(A)** Diagrams illustrating the ribosome binding regions of *TAS2* that were eliminated through CRISPR in *tas2-1*, *tas2-2*, and *tas2-3*. **(B)** Phenotypes of WT and *tas2-1*, *tas2-2*, *tas2–3* mutant seedlings after 12 days of growth on 1/2 MS medium. Scale bar = 5 mm. **(C)** The distribution of sRNA lengths, ranging from 18 to 26 nt, among WT, *tas2-1*, *tas2-2*, and *tas2–3* seedlings. The y-axis depicts the RPM of sRNAs of various lengths. **(D)** The IGV displays the abundance and phasing score of 21-nucleotide sRNAs generated at the *TAS2* locus in WT, *tas2-1*, *tas2-2*, and *tas2–3* plants. The radial graphs on the right illustrate the abundance of ta-siRNAs at various phases. Notably, the phasing scores for *tas2–2* and *tas2–3* did not surpass the threshold of 150, indicating the minimal ta-siRNA production in them.

The tertiary siRNA biogenesis is initiated by AGO1-bound secondary siRNAs, resembling the one-hit model where AGO1-bound 22-nt miRNA guides the cleavage. To further investigate whether the impaired tertiary siRNA biogenesis resulted from the depletion of ribosomes from the transcripts, we selected the ta-siRNA derived from *TAS2*, which follows the one-hit model, for subsequent analysis. We generated truncated mutants of *TAS2*, designated *tas2-1*, *tas2-2*, and *tas2-3*, respectively. These mutants were designed using CRISPR/Cas to delete varying lengths of the ribosome binding regions on *TAS2*, spanning nucleotides 269-374, 172-374, and 115-372, respectively (**Figure 5A**). DNA sequencing of the *TAS2* locus confirmed that nucleotide deletions occurred at the targeted positions as expected, verifying the successful creation of these mutants (**Supplementary Figure S2**).

During the initial developmental phase, the mutant lines cultivated on 1/2 MS medium exhibited no discernible growth phenotype differences from the WT, as illustrated in **Figure 5B**. This observation aligns with the function of *TAS2*-derived ta-siRNAs, which are involved in the immune response against fungal pathogens rather than in growth and development (Zhan and Meyers, 2023; Padilla-Padilla et al., 2024).

We first investigated the sRNA levels in seedlings of *tas2-1*, *tas2-2*, *tas2–3* mutants, as well as the WT. Analysis of the length distribution of the data revealed, as expected, that 24-nt sRNAs were most abundant within the 18-26-nt range, followed by 21-nt sRNAs (**Figure 5C**). Subsequently, we examined the abundance of all miRNAs, with particular focus on miR173, and confirmed that mutations in the *TAS2* locus did not affect miRNA expression (**Supplementary Figure S3**).

Subsequently, we performed a comprehensive analysis of the ta-siRNAs at the *TAS2* locus in all *tas2* mutants and WT plants. Concurrent with the truncation of the ribosome binding regions, sRNA abundance at *TAS2* exhibited a progressive decrease in *tas2-1*, *tas2-2*, and *tas2-3* (**Figure 5D**). Interestingly, *tas2–2* and *tas2–3* demonstrated significantly reduced phasing scores, with *tas2–3* exhibiting the highest phasing score below 150, failing to surpass the threshold (**Figure 5D**). The exception was *tas2-1*, which contains the shortest mutation. These observations suggest a critical role for the ribosome in ta-siRNA biogenesis. However, in contrast to our findings with the *tas3a/b/c* mutants, the deletion of the majority of ribosome binding regions in *TAS2* appears to substantially impair ta-siRNA production.

### 2.6 Absence of ribosome binding region promotes miRNA-guided cleavage

Given the observed decrease in sRNA abundance concurrent with the truncation of ribosome binding regions in *TAS2*, we sought to examine whether similar changes occurred at the level of intact transcripts in mutants. To this end, mRNA-seq analyses were conducted on the *tas2-1*, *tas2-2*, *tas2–3* mutants, and WT. The results revealed that the abundance of intact *TAS2* transcripts followed a pattern similar to that of sRNAs, exhibiting a progressive reduction in *tas2-1*, *tas2-2*, and *tas2-3* (**Supplementary Figure S4A, B**). RT-qPCR was used to detect the abundance of *TAS2* transcripts in *tas2–2* in the component of membrane associated polysome (MBP) as well, and it was found that the transcript abundance decreased more in MBP compared to the total cell lysis in the mutant, indicating that in the wild type, *TAS2* transcripts were mainly bound by MBP (**Supplementary Figure S4**).

**Figure 6:**
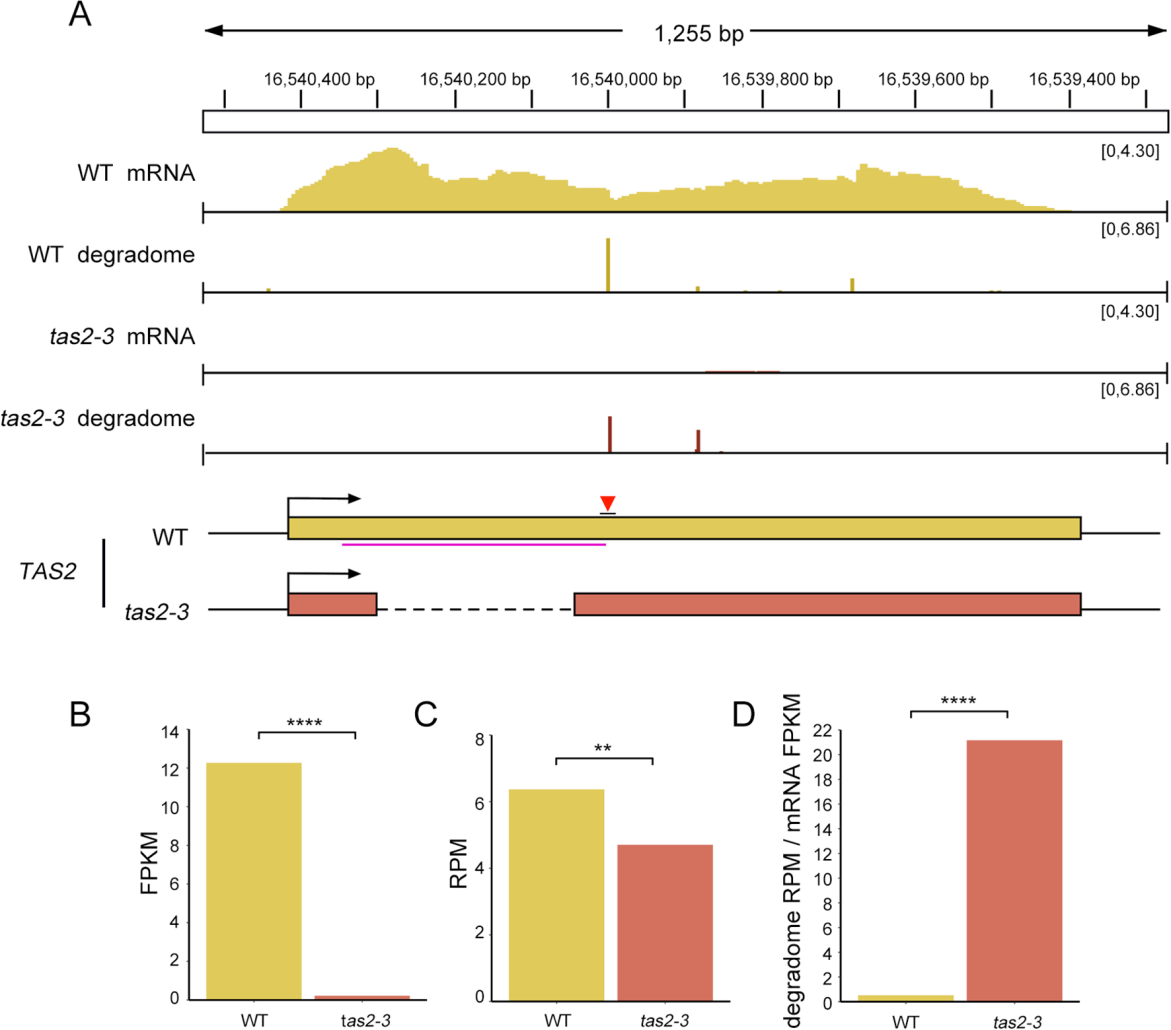
The efficiency of miR173-mediated cleavage of *TAS2* transcripts is substantially enhanced in the *tas2–3* mutant. **(A)** IGV visualization of the abundance of full-length *TAS2* transcripts and cleaved *TAS2* transcripts in WT and *tas2–3* mutant plants. **(B)** Full-length transcript abundance in FPKM in WT and *tas2-3*, with FPKM values representing the average of two replicates. *P*< 0.0001. **(C)** Abundance of transcripts cleaved by miR173-AGO1 in WT and *tas2-3.* The RPM is calculated based on the alignment of the 5’ end at the position chr2:16540000, with RPM representing the average of two replicates. Fisher’s exact test *P*=0.0017. **(D)** The efficiency of miR173-mediated cleavage on *TAS2* transcript in WT and tas3a/b/c mutant backgrounds is quantified as the ratio of abundance of cleaved transcripts **(C)** to that of full-length transcripts **(B)**. The efficiency of miR173-mediated cleavage of *TAS2* transcripts in the *tas2–3* mutant is significantly higher than in the WT. One-sided t-test *P* < 0.0001. ** represents P ≤ 0.01, *** represents P ≤ 0.001.

To examine the relationship between ribosome binding regions and phased siRNA biogenesis, this study focused on the mutant *tas2-3*, which exhibited the longest deletion and the lowest siRNA abundance at the *TAS2* locus. RNA-seq analysis revealed a significantly lower abundance of intact transcripts in *tas2–3* compared to the WT (**Figure 6A, B**, and **Supplementary Figure S4B**). This finding aligns with the sRNA-seq data, which indicated a low phasing score for *TAS2*-derived ta-siRNAs in *tas2-3*.

The reduction in unprocessed *TAS2* transcripts appears to be a crucial factor impeding ta-siRNA biogenesis. However, when compared to the tertiary siRNAs in *tas3a/b/c*, which maintained intact *TAS3a* transcripts yet displayed an almost complete absence of tertiary siRNAs, it suggests that additional mechanisms or stages in ta-siRNA biogenesis are also impacted.

To further investigate whether miR173-guided cleavage at the *TAS2* locus was impaired in mutants, we conducted degradome-seq analysis on *tas2–3* and WT. Surprisingly, we observed that the abundance of transcript cleavage by miRISC at the *TAS2* locus was comparable to that in WT (**Figure 6A, C**). Considering that the degradome generally reflects transcript abundance (Addo-Quaye et al., 2008), we hypothesized that the intact transcripts from the *TAS2* locus in *tas2–3* were not being degraded once transported to the cytoplasm. Consequently, the absence/reduction of ta-siRNAs might not be attributed to a deficiency of ta-siRNA precursors, as miR173-guided cleavage on *TAS2* transcripts appeared efficient in *tas2-3*. The significantly elevated miR173-guided cleavage efficiency in *tas2–3* further supported this hypothesis, as illustrated in **Figure 6D**. Integrating these findings with the observed reduction in *TAS2*-derived ta-siRNAs, we proposed that the removal of ribosome binding regions on *TAS2* enhanced the efficiency of miR173-guided cleavage of *TAS2* transcripts, although certain subsequent processes, such as dsRNA biogenesis and RNA duplex formation, are impeded. This impediment in downstream processes could also explain the loss of *TAS3a*-derived tertiary siRNAs in the *tas3a/b/c* mutants, suggesting that the integrity of ribosome binding regions is crucial for the complete biogenesis of ta-siRNAs and tertiary siRNAs.

## 3 Discussion

MicroRNAs are crucial regulatory non-coding RNAs that function both autonomously and non-autonomously. The biogenesis, trafficking, function, and degradation of miRNAs have been extensively investigated for decades, along with the corresponding subcellular localization of these processes (Zhan and Meyers, 2023). However, the subcellular location of ta-siRNA biogenesis remains less understood. Previous research has shown that both AGO1 and AGO7 co-fractionate with ribosomes, and miRNAs associate with ribosomes in a manner dependent on their target transcripts (Lanet et al., 2009). Although *TAS* genes are typically classified as non-coding genes, they contain ORFs that exhibit evidence of ribosome binding, as revealed by Ribo-seq analysis (Hou et al., 2016; Li et al., 2016). This observation has led to the hypothesis that ta-siRNA biogenesis may occur in association with ribosomes. Li et al. proposed that ribosomes contribute to ta-siRNA biogenesis by preventing transcripts from undergoing miRISC cleavage (Li et al., 2016). Indeed, while a substantial number of miRNAs have 22-nucleotide isoforms potentially capable of inducing ta-siRNA production, only a select few miRNAs actually facilitate ta-siRNA biogenesis *in vivo (* Li et al., 2016).

To further examine the relationship between ribosomes and ta-siRNA biogenesis, we conducted a series of sequencing analyses on *Arabidopsis* mutants *tas3a/b/c* and *tas2*, which exhibit deficiencies in ribosome binding at the *TAS3* and *TAS2* loci. The findings indicate that at the *TAS3a* locus in *tas3a/b/c*, which generates ta-siRNAs through the two-hit model, only the abundance of intact transcripts is diminished compared to the WT, while ta-siRNA abundance and miRNA cleavage efficiency remain unaffected. This observation is consistent with results from ribosome binding site-deleted *TAS3a* loci that accessed in *Nicotiana benthamiana*, where reduced transcript abundance is due to stability changes (Bazin et al., 2017). Notably, tertiary siRNAs derived from the *TAS3a* locus, which follows the one-hit model, were absent in the mutants. Sequencing and analysis were subsequently performed on the *TAS2* locus ribosome binding-deficient mutant *tas2*. The mutant with the most extensive deletion of ribosome binding regions, *tas2-3*, displayed low ta-siRNA abundance in sRNA-seq and reduced *TAS2* transcript abundance in mRNA-seq. However, degradome-seq revealed that *tas2–3* transcript abundance is comparable to the WT, while the efficiency of miR173-mediated cleavage on *TAS2* transcripts is significantly higher than in the WT. The production of ta-siRNAs in *tas2–3* is consistent with findings in maize *24PHAS_NO296*, where biogenesis follows the one-hit model. The deletion of ribosome binding regions does not appear to affect transcript stability but results in reduced phasiRNA abundance (Han et al., 2024).

**Figure 7:**
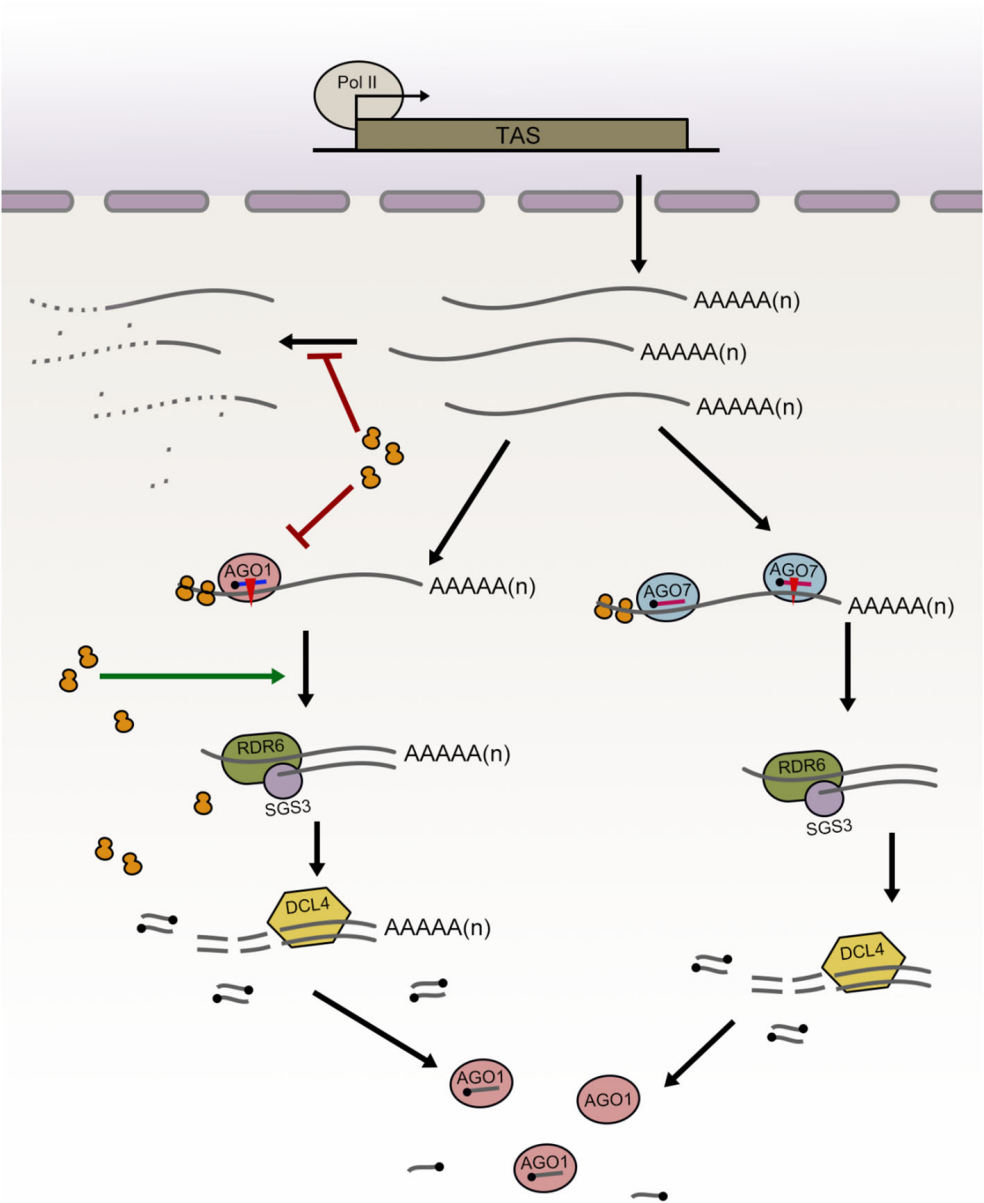
A proposed working model illustrating the distinct roles of ribosomes in the one-hit and two-hit ta-siRNA biogenesis models. In the one-hit model (left), ribosomes potentially inhibit the degradation of full-length *TAS* transcripts while simultaneously suppressing miRNA-targeted cleavage. Post-cleavage, ribosomal presence facilitates the entry of cleaved *TAS* transcripts into the ta-siRNA biogenesis pathway (encompassing dsRNA synthesis and DCL-mediated dsRNA cleavage). Consequently, when ribosome binding is compromised, *TAS2* transcript abundance decreases, miRNA cleavage efficiency increases, and sRNA abundance diminishes. In the two-hit model (right), ribosomes primarily function to inhibit the degradation of full-length *TAS* transcripts, potentially without significant involvement in other processes.

Based on these results, we propose a working model that integrates ribosomes and the two pathways of ta-siRNA biogenesis, as illustrated in **Figure 7**. Under normal conditions, cells require only a limited number of ta-siRNAs, preventing high-level production of potentially hazardous 22-nt siRNAs, while maintaining a substantial pool of *TAS* transcripts for rapid responses to stress or developmental signals. In the two-hit model of ta-siRNA biogenesis, ribosomes protect cytoplasmic *TAS* transcripts from degradation. Consequently, when the ribosome binding regions of *TAS3a* are deleted, the abundance of intact *TAS3a* transcripts decreases. However, the efficiency of miR390-guided cleavage remains unaffected, allowing for continued ta-siRNA generation from the *TAS3a* locus. In contrast, the role of ribosomes in the one-hit model is more complex. After *TAS2* transcripts are transported into the cytoplasm, ribosome binding contributes to their stability. However, upon entering the ta-siRNA biogenesis pathway, ribosome binding significantly reduces the efficiency of miRISC-mediated cleavage of *TAS2* transcripts. This may represent an alternative mechanism to prevent the overaccumulation of 22-nt ta-siRNAs. Following cleavage, a ribosome-rich environment facilitates RDR6-dependent dsRNA biogenesis and DCL4-mediated slicing. When the ribosome binding region is deleted, *TAS2* transcripts become unstable and more susceptible to degradation; the efficiency of miRISC-mediated cleavage on *TAS2* transcripts that enter the ta-siRNA biogenesis pathway is significantly enhanced; however, the truncated transcript fragments that are unable to undergo further processing are also degraded, leading to a decrease in final ta-siRNA abundance.

Given the disparities in ribosome function between the one-hit and two-hit models, we propose that the uncleavable miRISC binding to *TAS3* may assume certain ribosomal functions in maintaining mRNA stability. The miR390-*TAS3* pathway, an ancient molecular mechanism, is prevalent across dicotyledonous and monocotyledonous plants, including the earliest land plants such as mosses (Axtell et al., 2006). In these species, *TAS3* transcripts may undergo single or double cleavage, yet all exhibit conserved features, notably two miRISC binding sites, which constitute a key distinction between the one-hit and two-hit models. Prior research indicates that in *Arabidopsis*, the 5’ miRNA binding sites at the *TAS3* loci contain mismatches with miR390 at positions 9–11 nt, resulting in the uncleavability of the 5’ miRNA binding site. This uncleavable miRISC binding site may serve a crucial function. We suggest that the uncleavable RISC binding stabilizes *TAS3* transcripts. This hypothesis explains why, despite having similar proportions of ribosome binding region deletions, *tas3a/b/c* and *tas2–3* exhibit differences in their full-length transcript abundance.

## 4 Materials and methods

### 4.1 Plant materials and growth

*Arabidopsis thaliana* WT(accession Columbia-0) and CRISPR-Cas9-generated deletion mutants *tas3a/b/c* and *tas2-1*, *tas2-2*, and *tas2–3* were employed in this investigation. Seeds were germinated on 1/2 MS medium, maintained at 22°C under long-day conditions (16 hours of light and 8 hours of darkness). Following 12 days of cultivation, a subset of seedlings was transplanted into soil under long-day conditions in a plant growth chamber for phenotypic observation, while the remainder were harvested for sequencing and subsequent analysis.

The *tas2* and *tas3* mutants were obtained by genome editing of CRISPR/Cas9 using 2 single-guide RNAs (sequences of sgRNAs were listed in Supplementary **Supplementary Table S1**). The pHEE401 plasmid (Wang et al., 2015), serving as the Cas9-sgRNA expression vector, was digested by by BsaI restriction enzyme. Then constructs were introduced into Agrobacterium strain GV3101. The genetic transformation of *Arabidopsis thaliana* was carried out using the floral dip method (Clough and Bent, 1998). *tas3a/b/c* triple mutant was obtained through cross of *tas3a*, *tas3b* and *tas3c* single mutants.

### 4.2 Total RNA extraction and sRNA library construction

Total RNA was extracted from 12-day-old *Arabidopsis* seedlings using the TRIzol method (Takara, 9108). Small RNAs (sRNAs) of varying lengths were separated by 15% Urea-PAGE, and the 15-40bp bands were excised post-electrophoresis. The gel slices were soaked overnight at 4°C in 0.4M NaCl solution, followed by ethanol precipitation. The sRNA library construction was performed using the NEBNext Multiplex Small RNA Library Prep Set for Illumina (NEB, E7300S), adhering to the manufacturer’s protocol.

### 4.3 Analysis of sRNA-seq data and calculation of phasing score

We employed the pRNASeqTools v0.8 pipeline (available at https://github.com/grubbybio/pRNASeqTools) to analyze sRNA-seq data. The analysis commenced with the removal of adapter sequences (AGATCGGAAGAGC) and selection of reads sized 18-42nt using cutadapt v4.0 (Kechin et al., 2017) with the parameters ‘-m 18 -M 42 –discard-untrimmed –trim-n’. Subsequently, raw reads were aligned to the Araport11 genome using ShortStack v4.0.3 (Axtell, 2013b; Shahid and Axtell, 2014) with the parameters ‘–align_only –mmap u’. To quantify and compare read abundance across samples, the genome was divided into 100bp bins (or bins categorized according to annotations, such as miRNA, TE, gene, etc.). The read abundance for each bin was determined by calculating the number of reads with their 5’ ends falling into specific bins relative to the total number of reads, expressed as RPM. The methodology and formula for calculating the phasing score are referenced from Yang, Kun et al (Yang et al., 2018).

### 4.4 AGO1 IP and sRNA-seq

WT and mutant seedlings underwent treatment with cell lysis buffer (1M Tris-HCl, 5M NaCl, 10% NP-40, 1M MgCl2, 1M DTT, proteinase inhibitor cocktail (100x), RNase inhibitor) to extract total protein. Subsequently, AGO1 antibody (Agrisera, #AS09527) and Protein A dynabeads (Thermo, 10001D) were utilized to enrich AGO1 protein. Following this enrichment process, RNA extraction and construction of the small RNA (sRNA) sequencing library were conducted as previously described.

### 4.5 mRNA-seq and analysis

The RNA sequencing libraries for WT and mutant seedlings were constructed by Novogene. Initially, polyA-tailed RNAs were selectively enriched from the total RNA pool using oligo d(T) beads. These enriched RNAs were subsequently fragmented into random segments. Utilizing these segments as templates and dNTPs as building blocks, double-stranded cDNA was synthesized. Following purification, end repair, and adapter ligation, cDNA segments measuring 370-420bp were selected for amplification to create the RNA sequencing library. For mRNA-seq data analysis, pRNASeqTools v0.8 was employed. The process commenced with the removal of adapter sequences (AGATCGGAAGAGC) using cutadapt v4.0, followed by read alignment to the Araport11 genome with STAR v2.7.11a (Dobin et al., 2013), employing parameters ‘–alignIntronMax 5000 –outSAMmultNmax 1 –outFilterMultimapNmax 50 –outFilterMismatchNoverLmax 0.1’. Finally, featureCounts V2.0.6 (Liao et al., 2014) was applied to quantify the reads mapping to genes.

### 4.6 Degradome library construction, sequencing and analysis

The comprehensive protocol for constructing the degradome sequencing library is elaborated in Li, Yong-Fang et al (Li et al., 2019). Analysis of degradome sequencing data was conducted using pRNASeqTools v0.8. The process began with the removal of adapter sequences (AGATCGGAAGAGC) using cutadapt v4.0, followed by mapping reads to the Araport11 genome using STAR v2.7.11a with specific parameters: ‘–alignIntronMax 5000 –outSAMmultNmax 1 –outFilterMultimapNmax 50 –outFilterMismatchNoverLmax 0.1’. Subsequently, the bamCoverage function of deepTools v3.5.5 (Ramírez et al., 2014; Ramírez et al., 2016) was utilized to quantify the abundance of reads with 5’ ends aligned to specific loci, employing parameters: –skipNAs -bs 5 –minMappingQuality 10 –ignoreDuplicates –normalizeUsing CPM.

### 4.7 MBP isolation and RT-qPCR analysis of ribosome-associated edited *TAS2* transcripts

Seedlings (2 g) were finely ground into powder and resuspended in 7 mL of ice-cold MEB buffer (100 mM Tris-Cl [pH 7.5], 5 mM EGTA, 15 mM MgCl_2_, 5 mM DTT, 0.3 M sucrose, 2.5 U/mL SuperaseIN [Invitrogen], 50 µg/mL cycloheximide, 50 µg/mL chloramphenicol, and 1× proteinase inhibitor cocktail). After centrifugation, 200 µL of the supernatant was saved as the input control. The remaining supernatant was carefully layered onto a 20%/60% sucrose gradient column (2.5 mL per concentration; prepared in 100 mM Tris-Cl [pH 7.5], 5 mM EGTA, 15 mM MgCl_2_, 5 mM DTT, 20%/60% sucrose, 2.5 U/mL SuperaseIN [Invitrogen], 50 µg/mL cycloheximide, 50 µg/mL chloramphenicol, and 1× proteinase inhibitor cocktail). The gradient was centrifuged at 100,000 × g for 1 hour at 4°C using an SW41 rotor (Beckman Coulter). Following centrifugation, the green interface between the two sucrose layers was collected and diluted with 10 volumes of MEB buffer. The mixture was then centrifuged again at 100,000 × g for 30 minutes. The resulting pellet was lysed in 8 mL of PEB buffer (0.2 M Tris-Cl [pH 9.0], 0.2 M KCl, 0.025 M EGTA, 0.035 M MgCl_2_, 1% detergent mix, 1% polyoxyethylene (10) tridecyl ether, 5 mM DTT, 1 mM PMSF, 50 µg/mL cycloheximide, 50 µg/mL chloramphenicol, and 2.5 U/mL SuperaseIN) and further purified through a sucrose cushion (0.4 M Tris [pH 9.0], 0.2 M KCl, 0.005 M EGTA, 0.035 M MgCl_2_, 1.75 M sucrose, 5 mM DTT, 50 µg/mL cycloheximide, and 50 µg/mL chloramphenicol) by centrifugation at 50,000 rpm for 3 hours at 4°C using a Type 70 Ti rotor (Beckman Coulter). The MBP pellet was resuspended in resuspension buffer (0.2 M Tris-Cl [pH 9.0], 0.2 M KCl, 0.025 M EGTA, 0.035 M MgCl_2_, 5 mM DTT, 50 µg/mL cycloheximide, and 50 µg/mL chloramphenicol) and prepared for RNA extraction.

RNA was extracted from both the input and MBP fractions using the Trizol method, followed by DNaseI digestion to remove genomic DNA contamination. Reverse transcription and quantitative PCR (RT-qPCR) were performed using a one-step RT-qPCR kit (Takara, Cat. RR066A) according to the manufacturer’s instructions. Primer *tas2*-N-riboprotect and Primer *tas2*-C-cleaveage were designed to amplify the 5′ fragment upstream of the deleted ribosomal binding sites and the 3′ fragment downstream of the miR173 cleavage site, respectively, to assess the levels of edited *TAS2*–2 transcripts. The sequences of the qPCR primers are listed in Supplementary **Supplementary Table S2**. UBQ5 was used as an internal control for normalization, and the comparative CT (ΔΔCT) method was employed to quantify the qPCR results.

## Data Availability

The data generated and analyzed during this study have been deposited in the NCBI GEO under accession number: GSE295326.
