# Supplementary material for: Ribosomes binding to TAS transcripts buffer ta-siRNA biogenesis in Arabidopsis thaliana
Source: Front Plant Sci. 2025 May 19;16:1561041. doi: 10.3389/fpls.2025.1561041 (PMC12127299; doi:10.3389/fpls.2025.1561041)
Supplement: Supplementary file 1 [file Table1.docx]

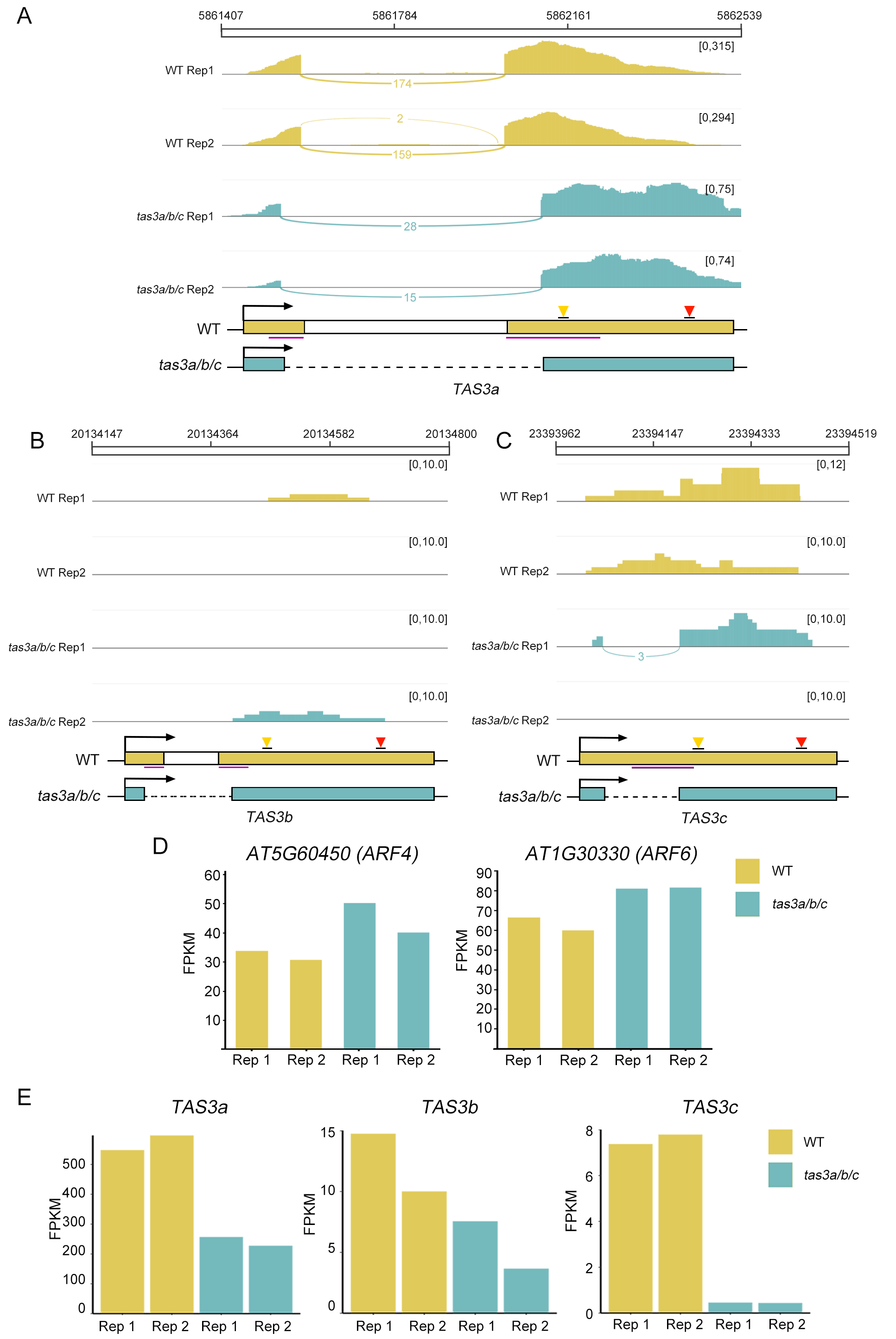


**Supplementary Figure 1**. mRNA-seq confirmed the deletion of the ribosome binding regions in *TAS3a* (A), *Tas3b* (B), and *Tas3c* (C) loci. The y-axis of the sashimi plot represents the read count. The numbers in arcs indicate junction read counts.

1. The mRNA abundance of known *TAS3*-derived tasiRNA targets *ARF4* and *ARF6* from the mRNA-seq. (E) The sRNA abundance (FPKM) at the *TAS3a*, *TAS3b*, and *TAS3c* loci in WT and *tas3a/b/c*. In comparison to *TAS3a*, the expression of sRNA is significantly lower at the *TAS3b* and *TAS3c* loci. Consequently, subsequent analysis disregarded sRNAs derived from these two loci.


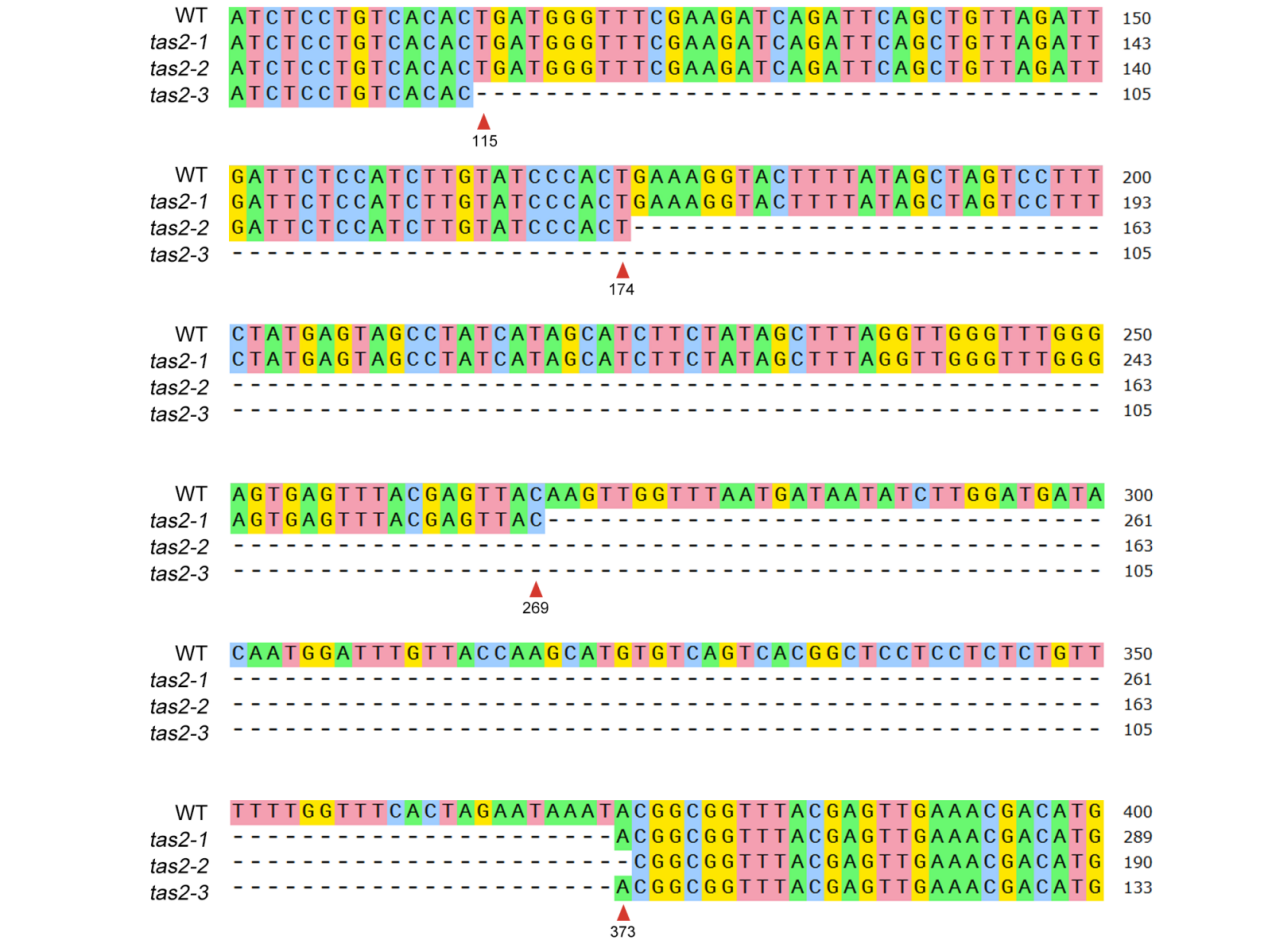


**Supplementary Figure 2.** Alignment of DNA sequencing results at the *TAS2* locus in *tas2-1*, *tas2-2*, and *tas2-3*. Triangular arrows with numbers indicate the base positions counting from the *TAS2* 5' end.


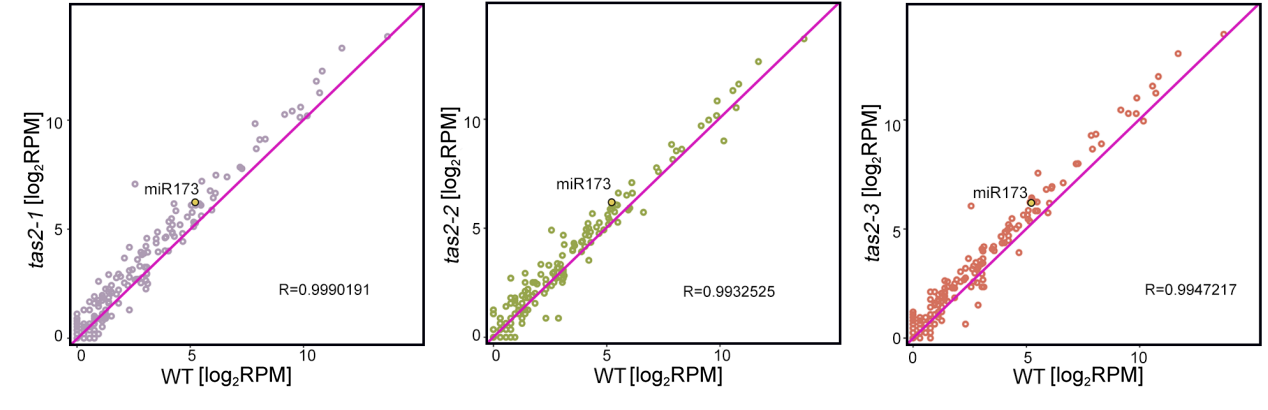


**Supplementary Figure 3.** miRNA expression profiles in *tas2-1*, *tas2-2*, and *tas2-3*. In all three scatter plots, the x-axis depicts the log_2_ (RPM) of miRNAs in the wild-type (WT), while the y-axis illustrates the log_2_ (RPM) of miRNAs in the respective mutant lines.


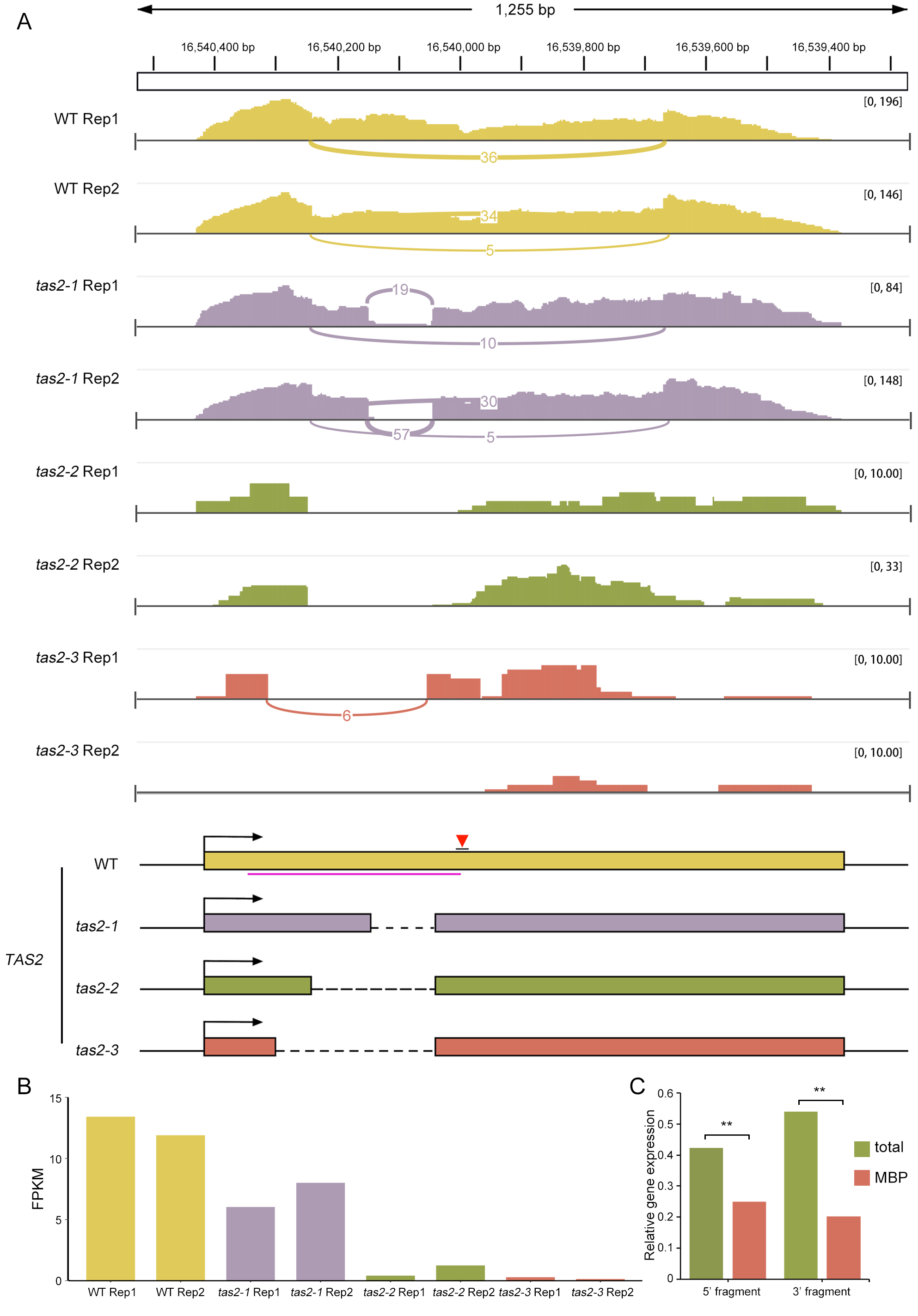


**Supplementary Figure 4.** decrease in mRNA abundance correlates with shorter ribosome binding regions. (A) The sashimi plot illustrates mRNA sequencing results of the *TAS2* transcript in wild-type(WT) and mutants *tas2-1*, *tas2-2*, and *tas2-3*. The y-axis denotes read counts, while colored numbers indicate junction read counts. Red triangles denote miRNA cleavage sites. (B) The graph displays mRNA abundance (FPKM) at the *TAS2* locus for WT and mutants *tas2-1*, *tas2-2*, and *tas2-3*. (C) qPCR results showed the changes of abundance of *TAS2* transcripts in total and MBP components in *tas2-2*. Both fragments upstream (5’) and downstream (3’) of the miRNA cleavage site decreased in total cell lysis of *tas2-2*, and even more on MBP. *P*-values for the 5' fragment and 3' fragment are 0.0084 and 0.0038, respectively, calculated by two-tailed Student’s *t* test. Two asterisks indicate a significant difference.
